# Developing a social prescribing local system in a European Mediterranean country: a feasibility study to promote active and healthy aging

**DOI:** 10.1186/s12913-021-07186-6

**Published:** 2021-10-27

**Authors:** A. Costa, J. Lopes, C. J. Sousa, O. Santos, A. Virgolino, P. Nogueira, A. Henriques, P. Seabra, C. Capitão, R. Martins, M. Arriaga, V. Alarcão

**Affiliations:** 1 Instituto de Saúde Ambiental (ISAMB), Faculdade de Medicina, Universidade de Lisboa, 1649-028 Lisbon, Portugal; 2 Nursing Research, Innovation and Development Centre of Lisbon (CIDNUR), Nursing School of Lisbon (ESEL), 1600-096 Lisbon, Portugal; 3 Católica Research Centre for Psychological, Family and Social Wellbeing, Faculdade de Ciências Humanas, Universidade Católica Portuguesa, Palma de Cima, 1649-023 Lisbon, Portugal; 4 Unbreakable Idea Research, Lda, 2550-426 Painho, Portugal; 5 Laboratório de Biomatemática, Instituto de Medicina Preventiva e Saúde pública, Faculdade de Medicina, Universidade de Lisboa, Avenida Egas Moniz, 1649-028 Lisbon, Portugal; 6 Directorate-General for Health, Health Literacy and Wellbeing Division, Alameda Dom Afonso Henriques, 1000-123 Lisbon, Portugal; 7 Centro de Investigação e Estudos de Sociologia, ISCTE—Instituto Universitário de Lisboa (ISCTE-IUL), Avenida das Forças Armadas, 1649-026 Lisbon, Portugal

**Keywords:** Social Prescribing, Primary health care, Community referral, Aged, Mixed-methods, On-the-job training

## Abstract

**Background:**

Social Prescribing (SP) is an innovative strategy to respond to the non-clinical health needs of the population. A Social Prescribing Local System (SPLS) can be defined as a set of joined community, health, and social organizations to foster SP-oriented activities. This study aimed to develop and assess the feasibility of an SPLS implemented in a Mediterranean country, to promote health and wellbeing and contribute to active and healthy aging.

**Methods:**

A mixed-methods approach was followed, including three sequential components: 1) Cross-sectional online survey targeting health professionals (HP) working in a primary health care cluster, Portugal’s southern region; 2) Pilot study implementing an on-the-job training program for HP, designed to meet identified training needs in the survey; 3) Focus group (FG) with the HP who participated in the pilot study, two individual interviews, with an elderly patient and a community provider for assessing the satisfaction with the pilot test.

**Results:**

Sixty-five HP completed the survey; of these, 13 completed the theoretical part of the on-the-job training program; and six (out of these 13) completed the full program. Five HP participated in the FG, one patient and one community provider were interviewed. The surveyed HP perceived as facilitators to implement SP: an automatic system of notifications to prompt the use of SP, contribute to patient satisfaction, human and community resources’ stability. The survey also highlighted barriers to SP implementation: length of appointments, shortage of human resources, data records confidentiality, low patient adherence rates, bureaucratic issues, time constraints, and financial costs. Participants were satisfied with the training. Identified SPLS implementation benefits were grouped into four dimensions (from the qualitative approach): gains for patients’ health and wellbeing, support for the health services, sustainability of the community resources, and HP’ professional satisfaction.

**Conclusions:**

Our study took the first steps towards the implementation of an SPLS. Findings reinforce that training HP in SP and on-the-job training seems feasible. This approach was well received and appears to represent a suitable and sustainable strategy. It can promote professional satisfaction, support health services, contribute to the stability of community resources, improve health and promote active and healthy aging.

## Background

Active and healthy aging implies the adoption of healthy lifestyles throughout the lifespan. Apart from clinical needs associated with the aging process, there are several non-clinical needs [1], which are often not adequately met by traditional health services [2].

Social Prescribing (SP) is an innovative strategy to respond to the non-clinical needs of the population [3]. The tertiary sector of local services develops prescribed activities within the community, enhancing patients’ community participation and social inclusion, with associated health and wellbeing benefits [4]. With SP, people’s health needs are addressed holistically. This approach incorporates multiple components (e.g., health promotion, motivation, and empowerment of patients), intervenients (e.g., patients, health professionals (HP), link workers, community providers), and outcomes (e.g., for the patient, HP, and health services) [5, 6]. This definition makes SP a complex intervention, being, therefore, important not for everyone involved, but also for the scientific community and policymakers, to understand how it can be developed, the underlying implementation processes, the mechanisms of intervention, and the context where it occurs [7].

A further challenge to SP interventions is to identify the available local community resources to establish collaborations between providers and health services and create suitable SP systems at the local level. A Social Prescribing Local System (SPLS) is a set of joined communities, health, and social organizations, in which professionals prescribe, join, streamline, monitor, and improve SP activities [8]. The SPLS should be adapted to each community, considering both the quantity and diversity of the supply of social resources and their functioning dynamics and interaction over time [9]. Indeed, to develop a sustainable SPLS in a community, three fundamental principles need to be considered: environmental sustainability, social and cultural sustainability, and economic sustainability [10]. These principles can ensure the health system’s sustainability and community support by providing a better quality of life, maintaining a good quality environment, and safeguarding natural resources [11]. Multiple studies have suggested that SP may have several potential benefits for the elderly population of SPLS [12], health systems [13], and communities [5].

Despite being an emerging approach, SP has been already successfully implemented by several professionals and communities. However, so far, there is a lack of consistent evidence about its effectiveness [3, 5, 14], partly due to diverse study designs and the shortage of studies with proper assessments [15]. Additionally, few published studies show the use of SP in populations culturally different from the United Kingdom, where SP is widely used, which hinders the assessment of how SP can play a role in different health care national services. Furthermore, the lack of structured and standardized learning programs for HP involved in SP interventions may have implications as these can cause differences in the compliance and performance of prescribers [16].

Social Prescribing has not been disseminated in Portugal [17]. Being a Mediterranean country with seasonal tourism and socio-cultural diversity, Portugal has adequate characteristics to implement an SPLS successful implementation: a very old population, an increasing patient-centered public health service [18], and a growing intersection of health and social community partners [19]. Social Prescribing may contribute to socioeconomic gains and the sustainability of community resources and community integration [20]. This study aimed to develop and assess the feasibility of an SPLS in a touristic region in a European Mediterranean country to promote active and healthy aging.

## Methods

**Fig. 1:**
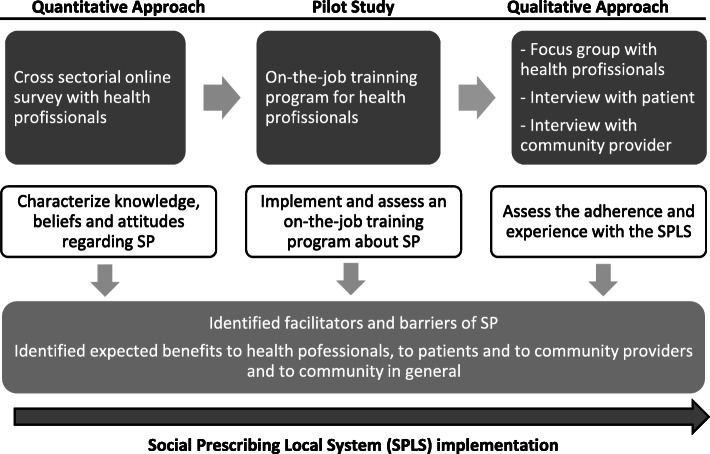
Study design

This study followed a mixed-method approach, including three sequential components (represented in Fig. 1):

- a cross-sectional online survey to characterize knowledge, beliefs and attitudes of HP, and to identify their training needs regarding SP;
- a pilot study to implement an on-the-job training program about SP for HP, using an SPLS, designed to meet the needs identified in the survey regarding training on SP;
- a qualitative study with focus groups and individual interviews to assess barriers and facilitators, adherence and experience with the SPLS by HP, and the receptivity and opinion of patients and community agents.

For clarity, hereinafter, we described each component separately. The study was developed in a tourist region of Algarve, in the southern coastline of Portugal, where 20.5% of the population is 65 years old or older, and 21.1% of these are living alone [21]. The region is an active municipality with diverse community resources, such as cultural and recreational associations, community allotments, making it a favorable location for creating an SPLS.

### Cross-sectional online survey: HP’ beliefs and attitudes towards SP

A cross-sectional study was conducted between July and August 2020, targeting HP working within a primary health care cluster (PHCC) from Algarve, Portugal. The PHCC was selected by convenience. All General Practitioners (GP) and community nurses from that PHCC (*n* = 359) were invited to participate (census approach). The email invitation was sent by the clinical direction of the PHCC with the link to the questionnaire. Three email reminders were sent by the PHCC secretary to minimize non-response rates.

The questionnaire was based on literature review and was designed in the LimeSurvey® platform (for self-administration). It comprised eight sections: 1) Sociodemographic and professional characterization of participants; 2) Empathy (based on Jefferson Scale of Physician Empathy (JSPE) [22, 23]); 3) Attitudes towards the therapeutic process (based on the “Patient-centered decision making” dimension from the Hypothesized Measurement Model of Interpersonal Processes of Care [24] which were then adapted by the research team for HP in general); 4) Knowledge about the concept of SP (based on Santoni et al. (2019) [16], which were then adapted by the research team for HP in general); 5) Perceptions about SP; 6) Expectations of patients’ adherence and receptivity to the implementation of an SP initiative; 7) Facilitators and barriers to the implementation of an SP program; and 8) Perspectives about SP training for HP. The questionnaire was pre-tested with 16 HP representing an heterogeneous sociodemographic and professional profile to ensure comprehension and consistency. The questionnaire was finalized and disseminated by email to all potential participants.

Questionnaires’ responses were analyzed using descriptive statistics. Absolute and relative frequencies for categorical variables and mean and standard deviations for numerical variables were used. Statistical analysis was performed using IBM SPSS 26.0 (IBM Corp. Released 2019. IBM SPSS Statistics for Windows, Version 26.0. Armonk, NY: IBM Corp).

### Pilot study: implementing an on-the-job training program

The pilot study was carried out from October to December 2020. Two health units from the PHCC were selected for convenience, and all HP (GPs and community nurses) were invited to participate in the pilot study. The HP that expressed their voluntary participation, being workers in a primary health care context that characteristically follows patients, providing clinical support throughout patients’ lifespan, aiming a healthy aging.

An on-the-job training program was designed to meet the needs identified by the survey. The project team developed support training program materials delivered to the participants, including a training program guide and a local community resources guide that participants should use to explore and update the providers to be involved in the constitution of the SPLS. This guide, as a dynamic working tool to be updated accordingly to the community dynamics, identifies the name and type of the social activity, the target population, the place and schedule, the price eventually implied in the community opportunities (free or the total amount), contacts, among other information about the community resources potentially available to establish collaborations with the health services. Health professionals may use this community-resources guide to choose and propose social activities to patients.

The training consisted of four hours of SP-related theoretical background (contents: European and Portuguese population health needs evolution; SP concepts; SP actors and process; How to create an SPLS; Evidence-based SP; Patients adherence to SP - promoting behavioral change and healthy aging), followed by 8 h of SP implementation real practice and 4 h of SP practice supervision made available by the project team to support the SP prescribing process. A further 11 h were allocated to participants’ SP organizational arrangements (data support, information flow, etc.), community contacts, and self-study. Participants used the community resources local guide, developed by the project team. They validated whether those community resources remained active, under what conditions, and if they were available to participate in the project as community providers. The training program was conducted by three team researchers with specific training skills (experience as academics and health professionals). Due to the coronavirus disease 2019 (COVID-19) pandemic, the training sessions took place online, through the Zoom® platform, and the second group only participated in the theoretical component of the training. Although there was a period of contact with community providers, for establishing the SPLS, patients were not involved due to the constraints of the pandemic. Participants answered an online questionnaire with ten questions about the general learning experience and satisfaction with the facilitator. The questionnaire was sent to participants by the PHCC secretary email and was available for self-application through LimeSurvey®. It assessed health professionals’ satisfaction with the training program (answers were given through a 5-point Likert scale and four open questions). The responses were analyzed using descriptive statistics. Absolute and relative frequencies for categorical variables and mean and standard deviations for numerical variables were used. Statistical analysis was performed using Microsoft Excel®.

### Qualitative study: the experience and satisfaction with the SP process for HP’, local providers, and one patient

The qualitative component of the study was held in December 2020. A semi-structured focus group was carried out with the HPs who participated in the pilot study. A semi-structured interview was also performed with a patient of the same primary health care unit that expressed his voluntary participation during the COVID-19 pandemic. Using video conference resources (Zoom® platform), both the focus group and the interview were conducted by a moderator and a co-moderator with experience in qualitative research, based on pre-defined interview scripts. Also, an individual interview was conducted with the community provider recently involved in the SPLS (museum).

The complete interview and focus group were audio-recorded, fully transcribed, and anonymized afterward. The obtained corpus (qualitative database) was analyzed independently by two researchers with experience in qualitative research, using a line-by-line open coding process, according to grounded theory principles (Charmaz [25]). After the independent open coding, interpretative triangulation was achieved by three members of the research team (two nutritionists and one psychologist). The qualitative analysis was performed with the software MAXQDA, version 2018.2.

### Ethical issues

The study was approved by the Ethics Committee of the Centro Académico de Medicina de Lisboa (CAML). The study also received a favorable appraisal from the Regional Administration of Health in Algarve.

In the quantitative study, collected anonymous data were compiled into a protected database, only accessible to the research team. The confidentiality of the information and the anonymity of participants was also ensured. Regarding the qualitative study, the audio was transcribed and anonymized, and the recorded material was later destroyed. All participants expressed their informed consent by signing the consent form.

## Results

The results from the three components of this study have been synthesized and analyzed together to assess the feasibility of developing and implementing an SPLS to promote active and healthy aging.

In the cross-sectional study, a total of 65 HP completed the online survey, of which 72.3% were nurses and 27.7% GPs. In total, 81.5% were women. The on-the-job training included 13 HP who completed the theoretical program while only six undertook the full program. The qualitative study about the experience and satisfaction with the SP process of HP, local providers, and patients included seven participants: one patient, one representative of a local community provider, and five HP. The sociodemographic and professional characterization of the participants is presented in Table 1.

**Table 1:** Sociodemographic and professional characterization of participants

	Participants (n)	Age group	Gender	Profession
**Survey**	65	25 to 75 years (66.2% between 35 and 54 years old)	53 (81.5%) women	Nurses: 47 (72.3%) GPs: 18 (27.7%)
**On-the-job training**	13 Team A: 6 Team B: 7	30 to 56 years	Team A: all women Team B: 2 men, 5 women	Team A: 5 nurses; 1 GP Team B: 6 nurses; 1 GP
**Focus groups and interviews**	HP: 5 Patient: 1 Community provider: 1	HP: 30–56 years Patient: 62 years Community provider^a^: NA	HP: all women Patient: men Community provider^a^: NA	HP: 4 nurses; 1 GP Patient: Electrician Community provider^a^: NA

*GP* general practitioner, *HP* health professionals, *NA* not applicable

^a^ The community provider was a museum, which responded to the interview as an institution and not as an individual person

Data regarding the feasibility of the SPLS are presented in Table 2.

**Table 2:** Feasibility of an SPLS

Survey	On-the-job training	Focus groups and interviews
• 64.6% had never heard of the concept of SP; • Empathy and acceptance of SP were significantly correlated positively; • 69.0% of the participants were unsatisfied with their knowledge of SP, 78.4% unsatisfied with how to apply it as a therapeutic resource, within their specific context; • 75.0% of the participants admit that they often or very often are confronted with non-clinical complaints; • More than 75% agree with the prescription of these activities: social activities; community physical activities; Artistic and creative activities; Technical/technological activities; Protection and personal development activities; Cultural deepening activities; Touristic activities; • 90.8% considered that receiving automatic notifications recalling SP will be a facilitator factor; • 95.4% view the contribution to improving patient’s satisfaction as a facilitator factor; • More than 85.0% of participants considered that the human and community resources’ stability were facilitators of SP.	• 53.9% of participants rated the training as “good” and the remaining 46.2% as “Excellent”; • After the training, all participants considered themselves able to be social prescribers; • Participants manifested the desire to replicate the training to other colleagues who expressed an interest in SP.	• Patient interview: ○ No knowledge of the concept of SP but recognized its relevance to both physical and mental health levels; ○ Physical activity, diet, intellectual activities, and even pharmacological therapy were recognized as important elements to promote and maintain health; ○ Many activities were identified as suitable in the scope of SP, namely: physical activity/exercise, traditional games, outdoor activities adapted to the local community (fishing, beach activities), among others. • HP’ Focus Group: ○ Suggested activities: dance and ballet classes; trails, walks, runs; yoga classes, pilates; sewing activities; picnics with patients and HP; swimming pools; ○ Positive aspects of the pilot study: good receptivity; Recognition of great potential; Potential patients´ satisfaction; ○ General satisfaction with the training program, and suggestions to replicate it to expand the number of prescribers, as other colleagues showed interest in collaborating with SP. • Community Provider Interview: ○ Provider showed interest, availability, and enthusiasm regarding the collaboration with SP.

*SPLS* social prescribing local system, *SP* social prescribing, *HP* health professionals

Regarding the online survey, data show that more than half (64.6%) of the participants were unfamiliar with the concept of SP. Receiving automatic notifications recalling SP, the perception that SP can improve patient satisfaction, and the stability of human and community resources, were identified by the HP as facilitators for the implementation of SP.

All participants in the on-the-job training program rated it as “good” or “excellent” and considered that they could work as Social Prescribers after the training. In the qualitative study, the patient identified activities suitable for prescribing adapted to the geographical location. The HP recognized the great potential of SP, and the community provider showed interest, availability, and enthusiasm regarding the collaboration.

Collected data also revealed barriers to the implementation of an SPLS (Table 3). Main identified barriers were: the length of appointments, as they are already long to include a new approach; human resources between HP communities; data records confidentiality; low patient adherence rates; bureaucratic issues; time constraints of patients; and possible financial costs.

**Table 3:** Identified barriers to SPLS implementation

Survey	On-the-job training	Focus groups and interviews
• The length of consultation was identified as a barrier to HP’s engagement with SP by 32.3% of the participants; • Human resources that ensure mediation between HP and the community; • Aspects regarding confidentiality of patient data records that could be accessed by other entities involved in SP.	• Expected low patient adherence rates.	• HP’ Focus Group: ○ Limited activity offered (especially due to COVID-19 pandemic); ○ Bureaucratic issues: very long waiting times when connecting with providers. • Patient interview: ○ Time required for the patient to dedicate to the activities prescribed; ○ Possible associated financial costs.

*SPLS* social prescribing local system, *HP* health professionals, *SP* social prescribing

The possible benefits of implementing an SPLS were grouped into four principal dimensions according to the potential beneficiaries’: improvements for patients’ health and wellbeing, support for the health services, sustainability of the community resources, and HP’ professional satisfaction.

Regarding patient benefits, as presented in Table 4, data collected in the three components of this study showed an agreement that SP benefits the patients’ health and wellbeing.

**Table 4:** Benefits to patients with implementation of an SPLS

	Survey	On-the-job training program	Focus groups and interview
**Benefits to patients**	• 92.3% of participants agreed that SP could benefit patients’ health and wellbeing.	• Training program participants fully agreed that SP can benefit patients’ health and wellbeing, contributing to healthy aging.	• HP’ Focus Group: ○ The benefits pointed out were: opposes sedentary lifestyle, improves mental health and wellbeing, and reduces the use of anti-depressives and anxiolytics. They also indicated that SP could respond to the need for socializing caused by isolation. ○ Based on the experience during the pilot study, HP shared that patients were thankful for the concern, additional attention, and opportunity to participate in SP.

*SPLS* social prescribing local system, *SP* social prescribing, *HP* health professionals

When asked in the survey about the profile of patients considered to be more likely to join the SP initiative, most HP (83.9%) considered that being a woman would be the characteristic most prevalent in adherents.

Concerning the optimization of health services with the development of an SPLS (Table 5), the majority of survey respondents (78.5%) considered that it would benefit their health care unit, (e.g., reduce the number of unscheduled appointments; and benefit the relationship between HP and patients). In the on-the-job training, participants pointed out that SP is better suited to address patients’ non-clinical care needs than traditional approaches. In the focus group, it was suggested that implementing an SPLS could improve the support available for mental health and that the potential to reduce the affluence to the health services could reduce the overload of HP. The possibility of collaborating with the city council and community was also considered a benefit for patients and the community.

**Table 5:** Optimization of health services, community development, and professional satisfaction with the development of an SPLS

	Survey	On-the-job training program	Focus groups and interview
**Optimization of health services**	• 78.5% considered that SP would benefit their health care unit; • 72.3% considered that SP could reduce the number of unscheduled appointments; • 80.0% considered that SP could benefit the relationship between HP and patients.	• During the training program, participants highlighted SP as a care quality improvement since it is better suited to address patients’ non-clinical care needs than traditional approaches.	• HP’ Focus Group: ○ For the health care unit, the benefits identified were: increasing the options/tools offered to patients, restoring the lack of psychologists, and improving mental health support. The possibility to articulate with the city council and community would also benefit patients and the community; ○ Reducting the affluence to the health services of people who only need to socialize, could reduce the overload of HP.
**Community development**	• 87.7% agreed that SP would benefit the local community in terms of social cohesion.	• Training program participants considered SP a very interesting approach that could benefit community health and development.	• Patient interview: ○ Organizations were identified to be involved in creating a partnership network, such as sports grounds, gyms, and urban gardens, although, regarding the latter, the interviewee raised doubts about the patient’s acceptance; ○ The patient suggested articulating with the City Council, Hospital Management, or Regional Health Administration. • Community Provider Interview: ○ Institutional benefits interpretation, enhancement, and promotion of the history and patrimony of the county; promotion of museum spaces, cultural education, and the creation of new audiences; contribution to the museum’s mission creating experiences of attraction and curiosity; provision of new knowledge through memories, identities, and territories.
**Professional satisfaction**	• 82.1% admitted that SP would improve their effectiveness.	• Motives for greater professional satisfaction with SP: ○ Being capable to better attend to patient non-clinical needs; ○ Diversify their daily health care activity; ○ Deepen their relationship with community providers.	• HP Focus Group: ○ Identified motivations to participate in the project: social aspects, namely working with people in the community; the pleasure of working with the target population’s age group; the opportunity to respond to the patients’ social isolation; the possibility to prescribe more than medication; and the belief in the SP potential. ○ Added value for HP: the improvement of the relationship with the patient; the increased perception of competence; and the consequent professional achievement, for providing better health care.

*SPLS* social prescribing local system, *SP* social prescribing, *HP* health professionals

Regarding community development with the implementation of an SPLS (Table 5), most participants in the survey (87.7%) agreed that SP would benefit the local community in terms of social cohesion, and, during the on-the-job training, it was underlined that implementing an SPLS could improve the health and development of the community. In the interview with the community provider, promoting the museum spaces and cultural education was identified as a benefit, by creating new audiences and providing new knowledge through memories, identities, and territories.

Regarding the professional satisfaction associated with developing an SPLS (Table 5), most HP who participated in the survey (82.1%) believed that SP would improve their and deepen their relationship with community providers. In the focus group, the participants recognized that SP could improve the relationships with patients and increase the perception of competence and professional achievement by providing better health care.

## Discussion

This feasibility study used a mixed-methods approach to develop an SPLS and assess its implementation to promote healthy aging in a touristic region. A self-administrated online questionnaire was developed to assess the knowledge, beliefs, and attitudes of HP towards SP. We identified that HP lacked training in SP and that this strategy is well accepted and considered relevant by all involved actors. The design of the study was discussed with the teams of the two health units involved and taking into account their organizational structure, GPs and nurses were available to participate without a link worker. Furthermore, during the pandemic context of the study implementation, not involving an additional person seemed to be a secure choice. Next, within the pilot study, the research team developed a training program to respond to the identified needs. Finally, using a qualitative approach, we identified barriers, facilitators, and assessed the acceptance of the project.

The study findings show that SP is relatively unknown to the Portuguese HP. Most participants who answered the online questionnaire were unfamiliar with the concept of SP, as well as the interviewed patient. After some *prompts*, the HP recognized its relevance and potential to improve mental health and wellbeing. However, general dissatisfaction with an unawareness on applying SP as a therapeutic resource within the health context was observed, underlining the eagerness for learning more about SP.

Some authors have previously identified factors that facilitate the implementation of an SPLS [9]. Our findings show that, from the perspective of the HP, the main facilitators are 1) reception of automatic notifications recalling SP; 2) perception that SP will contribute to improving patient’s satisfaction; and 3) human and community resources’ stability. These facilitators underline the importance of having human resources/connector roles (such as link workers), highlighted by Tierney et al. [6], and the importance of implementing an SPLS to assure the sustainability of local resources, promoting a synergic interaction between the patient, health services, and community [26]. In fact, although some SP models dispense the link worker, choosing to directly link HP with the community providers, as was the case in this study, the recognition of the importance of the link worker participation for the receptivity and engagement of users in the SP activities prescribed is a growing trend in the literature [27] . It seems that SP models work better when link workers are involved [27]. The results from the on-the-job training suggest a favorable attitude towards the implementation of an SP intervention. The HP expressed satisfaction and interest to learn more, and they suggested replicating the training, to expand the number of prescribers. These findings show that on-the-job training seems to be a good approach to implement an SPLS and develop non-pharmacological interventions in response to non-clinical needs [28]. Developing an SPLS in an evolutionary way, involving all agents, is also suggested [28].

The interviewed patient identified relevant activities in the local area, such as fishing or beach-related activities. It was exciting to note that the patient suggested typical activities relevant to the local reality because this idea was based on an SPLS. Moreover, being a touristic region in a Mediterranean country, it is important to monetize pre-existing resources to contribute to community sustainability [29]. SP has a great potential to articulate with tourist activities integrated within a local prescribing system that co-exists with the growing tourist phenomenon. The SP in a tourism context can promote community resources by involving touristic activities, improve the health and wellbeing of the elderly, reduce the costs to the health service, and contribute to the stability and sustainability of health and community resources. In recent decades as the tourism phenomenon was growing, touristic activities have been gradually rethought and reshaped in order to promote tourism as a factor of community cohesion and development. This created favourable conditions for stimulating innovation and entrepreneurship, even in the health and social care. Like SP activities, today’s touristic activities are more and more concerned with communities’ sustainability, and the preservation of their cultural and natural heritage, and local identity. Furthermore, the implementation of a SPLS in these regions allows to take advantage of and monetize infrastructures used by tourists in the high season, during the remaining months of the year, contributing to its sustainability [30, 31]. Understandably, SP will always depend on the interaction of many complex factors, such as the health services, community, and patients [32].

From the community services perspective, the interviewed community provider showed interest, availability, and enthusiasm regarding the collaboration with the SPLS. These are promising findings as they show that SP benefits the patients directly and the communities by encouraging the patients to connect with the local providers [26].

The data collected also highlights some barriers to the implementation of an SPLS. The main barriers found were aligned with the literature [9] and were, related to time constraints, either of HP regarding the appointments length (being hard to have time to integrate the role of social prescribers into HP agendas’) and the patients regarding their availability to adhere to the activities [9, 33]. Other identified barriers were lack of human resources between HP and communities, aspects regarding confidentiality of patient data records, low patient adherence rates, bureaucratic issues, and possible financial costs. Some of these barriers have been identified in previous literature [9].

Regarding the profile of patients considered to be more likely to adhere to SP successfully, the majority (83,9%) of HP considered that being a woman would be the most prevalent characteristic in facilitating adherence, which is in line with the literature [15]. This may be related to the perception of HP about the difficulty of men in seeking help about their emotional needs and help support compliance [34].

Concerning optimization of health services with the development of an SPLS, the majority (78.5%) of HP that answered the online questionnaire considered that it would benefit their health unit, agreeing that it could, for example, reduce the number of unscheduled appointments and improve the relationship between HP and patients. The participant HP highlighted that SP is better suited to address patients’ non-clinical care needs than traditional approaches and suggested that implementing an SPLS can improve mental health support [14]. Another benefit identified for SP was its potential to reduce the affluence to health services patients need to socialize [14].

Concerning community development with the implementation of an SPLS, participants agreed that SP would benefit the local community in terms of social cohesion. Authors have reported that implementing an SPLS enhances health status and community development [35]. The community provider pointed out the benefits of promoting their spaces, cultural education, creating new audiences, and providing new knowledge through memories, identities, and territories.

Professional satisfaction with the development of SPLS findings reflects that most (82.1%) believed that SP would make their practice more effective, considering they will better attend to non-clinical patient needs. The HP recognizes this approach value as it will improve their relationship with patients, increase the perception of competence, and consequent professional achievement, by providing better health care. Regarding the quality of the relationship with patients, the literature supports those relationships with equal distribution of power contribute to the professional self-efficacy perception and enhances patients’ adherence to therapeutic strategies and compliance to the HP’ guidance [36].

### Strengths and limitations

This study was developed during the COVID-19 pandemic, which implied less availability of participants, lower participation rate, and greater difficulty in establishing social partners. Given the restrictive context, a large part of the community resources had been severely reduced or suspended, and, consequently, only one social partner was identified as available. There were also limitations to having patients carry out the activities, precluding the assessment of patients’ activities, challenging the implementation of the SPLS as predicted. Despite these constraints, we trained HP, identified community resources, and activated community providers, as well as the connections with the health care unit. As it was not possible to carry out the training and focus groups in person, we carried them out online, via Zoom®, and obtained excellent feedback. All stakeholders were receptive and enthusiastic to continue the partnership. Our results show that SP could offer additional value given the current situation as it could be offered to patients as an additional intervention to mitigate the social isolation and loneliness related to the pandemic.

### Future recommendations

For the elderly populations, SP is of increased importance, with some evidence of improvements in individual health, interaction with others, institutions, and society [37, 38]. Our results are in line with previous literature that shows that SP is promising as a procedure in health, which can contribute to disease prevention and improvement in health systems responding to the priority societal challenge of demographic change, health and wellbeing [39].

In Portugal, as in most countries, health professionals job descriptions are clear and regulated, however other participant in the SP process are not. Further studies considering quality measures and role competencies of other participants involved in the SP process, such as community providers and link workers, are still needed.

Due to the enthusiasm and interest shown by the HP and providers and the positive results obtained from this study, a new project should be carried out. However, considering the importance of link workers for enrolment, engagement and adherence of SP users, their participation should be taken into account in future projects targeting SP. Furthermore, the next steps of the project must strengthen the community involvement [9] and perspectives and we would also like to expand this intervention to other health practices nationally and take our training to more HP. We would also like to expand this intervention to other health practices nationally and take our training to more HP. This approach can also be reproduced in other regions contributing to the development of SP in other regions.

## Conclusion

Our findings reinforce the importance of training in SP. On-the-job training seems to be an adequate choice when introducing SP in GP’s and community nurses’ daily practice. It can improve professional satisfaction, support the health care unit’s continuous improvement, provide conditions for health and social sectors to approach and work together, and widen therapeutic opportunities for a growing population of patients with non-clinical needs. Integrating SP in the practices of HP seems to contribute to the feeling of greater complementarity in the performance of their profession. We found productive ground for the implementation of the SPLS. It is a region where an SPLS did not exist, and we have created the initial conditions for its implementation and future development.

## Data Availability

The datasets used and/or analysed during the current study are available from the corresponding author on reasonable request.

## Abbreviations

CAML: Centro Académico de Medicina de Lisboa
COVID-19: Coronavirus Disease 2019
FG: Focus group
GP: General Practitioners
HP: Health Professionals
JSPE: Jefferson Scale of Physician Empathy
PHCC: Primary Health Care Cluster
SP: Social Prescribing
SPLS: Social Prescribing Local System
